# First In Vivo Insights on the Effects of Tempol-Methoxycinnamate, a New UV Filter, as Alternative to Octyl Methoxycinnamate, on Zebrafish Early Development

**DOI:** 10.3390/ijms24076767

**Published:** 2023-04-05

**Authors:** Elisabetta Damiani, Fiorenza Sella, Paola Astolfi, Roberta Galeazzi, Oliana Carnevali, Francesca Maradonna

**Affiliations:** 1 Department of Life and Environmental Sciences, Polytechnic University of Marche, 60131 Ancona, Italy; 2 Department of Science and Engineering of Materials, Environment and Urban Planning, Polytechnic University of Marche, 60131 Ancona, Italy

**Keywords:** embryo development, developmental toxicity, ER stress, *Danio rerio*, endocrine disruptors, bone development, UV filters

## Abstract

The demand for organic UV filters as active components in sunscreen products has rapidly risen over the last century, as people have gradually realized the hazards of overexposure to UV radiation. Their extensive usage has resulted in their ubiquitous presence in different aquatic matrices, representing a potential threat to living organisms. In this context, the need to replace classic UV filters such as octyl methoxycinnamate (OMC), one of the most popular UV filters reported to be a potential pollutant of aquatic ecosystems, with more environmentally friendly ones has emerged. In this study, using zebrafish, the first in vivo results regarding the effect of exposure to tempol-methoxycinnamate (TMC), a derivative of OMC, are reported. A comparative study between TMC and OMC was performed, analyzing embryos exposed to similar TMC and OMC concentrations, focusing on morphological and molecular changes. While both compounds seemed not to affect hatching and embryogenesis, OMC exposure caused an increase in endoplasmic reticulum (ER) stress response genes, according to increased *eif2ak3*, *ddit3*, *nrf2*, and *nkap* mRNA levels and in oxidative stress genes, as observed from modulation of the *sod1, sod2, gpr*, and *trx* mRNA levels. On the contrary, exposure to TMC led to reduced toxicity, probably due to the presence of the nitroxide group in the compound’s molecular structure responsible for antioxidant activity. In addition, both UV filters were docked with estrogen and androgen receptors where they acted differently, in agreement with the molecular analysis that showed a hormone-like activity for OMC but not for TMC. Overall, the results indicate the suitability of TMC as an alternative, environmentally safer UV filter.

## 1. Introduction

**Chart 1:**
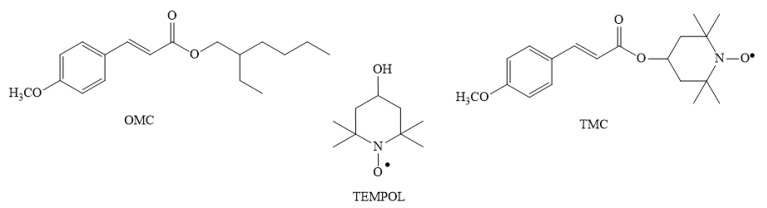
Chemical structures of OMC, TEMPOL, and TMC.

Ultraviolet (UV) filters present in sun care products play a pivotal role in preventing skin damage caused by overexposure to a broad spectrum of UV radiation that reaches the Earth’s surface [1]. However, increasing concerns have recently been raised regarding UV filters, as well as other cosmetic and personal care product additives, as several studies have shown that they can cause a series of adverse effects in animal models, spanning from developmental and neurological disorders [2,3,4] to reproductive impairment [5,6,7,8], including decreased semen [9,10] and oocyte quality [11], as well as transgenerational effects [12]. To date, one of the most used UV filters is octyl methoxycinnamate (OMC), also known as ethylhexyl methoxycinnamate (EHMC) or octinoxate, which, over the years, has been used worldwide in more than 90% of commercially available sunscreens and cosmetic products [13]. OMC toxicity has been documented, both in vitro and in vivo, in different animal models, either vertebrates or invertebrates [14,15,16,17,18,19]. Moreover, recently, in medaka, its transgenerational effect has also been stated [20], while, in zebrafish, parental exposure reduced the hatching rate and offspring growth [21]. Additional studies have reported a dose-dependent developmental toxicity in zebrafish embryos, which exhibited pericardial edema, a decrease in heartbeat rate, scoliosis, and tail malformation [13]. It has also been demonstrated that OMC exposure lowers the thyroid hormone levels in both adult males and larvae by activating different molecular pathways [22]. OMC exposure in larvae can also influence the transcription of genes related to neurotoxicity, whereas, in adults, it causes nephrotoxicity [22], stress and alters the antioxidant response [17]. Considering the state-of-the-art and following Hawaii’s recent ban, along with other countries, on selling sunscreen containing the UV filters oxybenzone and OMC [23], the pharmaceutical and cosmetic industries have been encouraged to review their formulations, substituting the above-mentioned UV filters with new ingredients and offering safer, environmentally friendly sunscreens. In this regard, in 2006, a new UV filter named 2,2,6,6-tetramethyl-piperidin-4-yl-methoxy cinnamoyl-1-oxyl, abbreviated as TMC, was synthesized from OMC. TMC derives from the UV-absorbing compound OMC, where the ethylhexyl moiety was replaced with an antioxidant compound, the nitroxide radical 4-hydroxy TEMPO (TEMPOL) (Chart 1) [24,25]. The effects of TMC on living organisms have never been investigated; therefore, evaluating the aptness of TMC as a safer sunscreen filter by testing its effects on animal models could thus be extremely valuable, and to this aim, zebrafish (*D. rerio*) could represent an excellent experimental model. This fish presents certain features that makes it unique in the fields of both molecular and developmental biology, including the large number of daily offspring, rapid embryonic development, and transparency that allows early organogenesis visualization and, last but not least, this organism and humans share a high degree of synteny [26]. Noteworthy, zebrafish also possess an antioxidant system sharing high homology with the mammalian one and a set of enzymes counteracting oxidative stress effects [27]. Thus, in this study, *Danio rerio* embryos were exposed to TMC and OMC in the range of OMC concentrations detected in the environment [13], and their effects on early development were analyzed and compared in order to view TMC as a possible candidate alternative to OMC. In this context, in fact, an in silico study has been recently published reporting evidence on the suitability of some chemical compounds as OMC alternatives [28]. The preliminary computational results are also reported here in order to support this hypothesis; in particular, the potential binding and affinity for TMC vs OMC has been evaluated by molecular docking calculations to estrogen and androgen receptors.

## 2. Results

### 2.1. Effect on Development, Hatching, and Growth Parameters

#### 2.1.1. Effect on Embryo Morphology

**Figure 1:**
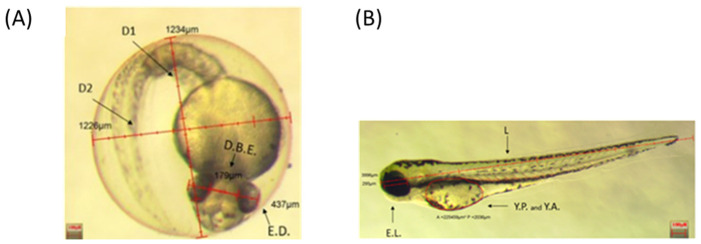
Inserts show a representative 48 hpf embryo (**A**) and 72 hpf larva (**B**), displaying the analyzed parameters. D1: Eye Diameter 1; D2: Eye Diameter 2; D.B.E.: Distance between eyes; E.D.: Eye diameter; E.L.: Eye length; Y.P.: Yolk perimeters; Y.A.: Yolk area; L: body length. Scale bar 100 µm.

To verify the possible interference of the two chemicals on the early developmental phases, the embryo and larvae were observed daily, starting just prior to hatching and using a stereomicroscope at 48, 72, and 96 h post-fertilization (hpf). At 48 hpf, the two eye diameters (D), the eye distance (E.D.), and the distance between the eyes (D.B.E.) were checked. At 72 and 96 hpf, the length of the larvae (L), the length of the eyes (E.L.), the yolk area (Y.A.), and its perimeter (Y.P.) were recorded. Figure 1A,B show representative images and details regarding the morphological parameters.

As shown in Table 1, at 48 hpf, no morphological differences were observed among the embryos exposed to the UV filters and in the control group. No differences occurred either among the embryos exposed to the three OMC and TMC concentrations.

Similarly, at 72 (Table 2) and 96 (Table 3) hpf, no morphological differences were observed among the control and exposed larvae.

#### 2.1.2. Effects of Treatments on Hatching Rate

**Figure 2:**
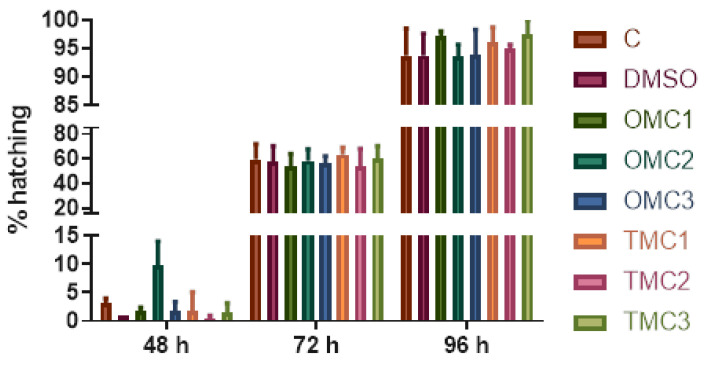
Hatching rate in experimental embryos/larvae at 48, 72, and 96 hpf.

The hatching rate was monitored daily starting from 48 hpf. At 48 h, in OMC2-exposed embryos, the hatching rate was higher, although not to a significant extent with respect to all the other experimental groups. At 72 hpf, the hatching rate reached 60% and was similar in all groups. At 96 hpf, more than 90% of the embryos were hatched in all groups, and no statistical significances were observed among the experimental fish (Figure 2).

#### 2.1.3. Alcian Blue–Alizarin Red Double Staining

**Figure 3:**
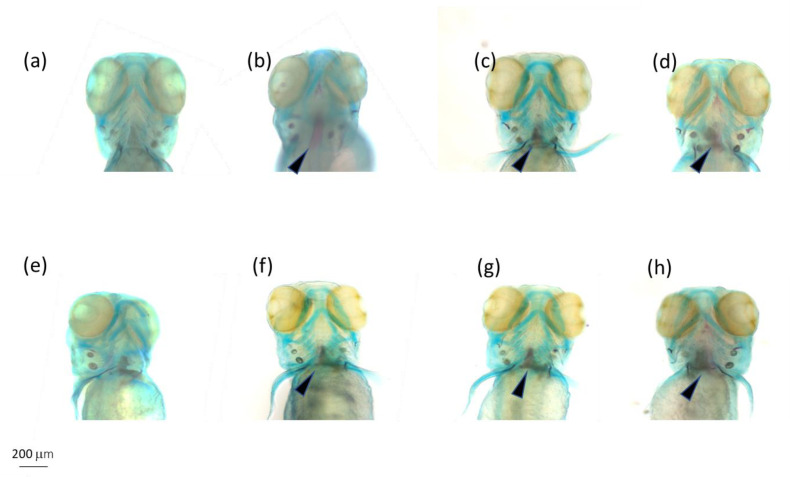
Alcian blue–Alizarin red double staining of the head skeleton (dorsal view) of 96 hpf larvae. (**a**) CTRL; (**b**) OMC1; (**c**) OMC2; (**d**) OMC3; (**e**) DMSO; (**f**) TMC1; (**g**) TMC2; (**h**) TMC3 (1 = 0.05 mM, 2 = 0.01 mM, and 3 = 0.005 mM). Arrows indicate the basioccipital articulatory process (BOP). Scale bar = 200 mm.

The double staining technique allows the simultaneous staining of different tissue types. Alcian blue is used to stain acidic polysaccharides such as glycosaminoglycans in cartilages and other body structures, while alizarin red reacts with calcium, thereby helping in the diagnosis of calcium deposits and, thus, is commonly used to mark bone tissue. Fish were stained at 96 h, a good time point to monitor cartilage [29]. Images obtained with the optical microscope show that, according to the developmental stage, the head skeleton presents mainly cartilaginous structures. However, it is possible to notice that larvae exposed to every OMC (Figure 3a–d) and TMC (Figure 3e–h) concentration already show signs of the calcified basioccipital articulatory process (BOP), thus suggesting a possible acceleration in development.

### 2.2. Analysis of Molecular Targets Involved in Oxidative Stress Response

**Figure 4:**
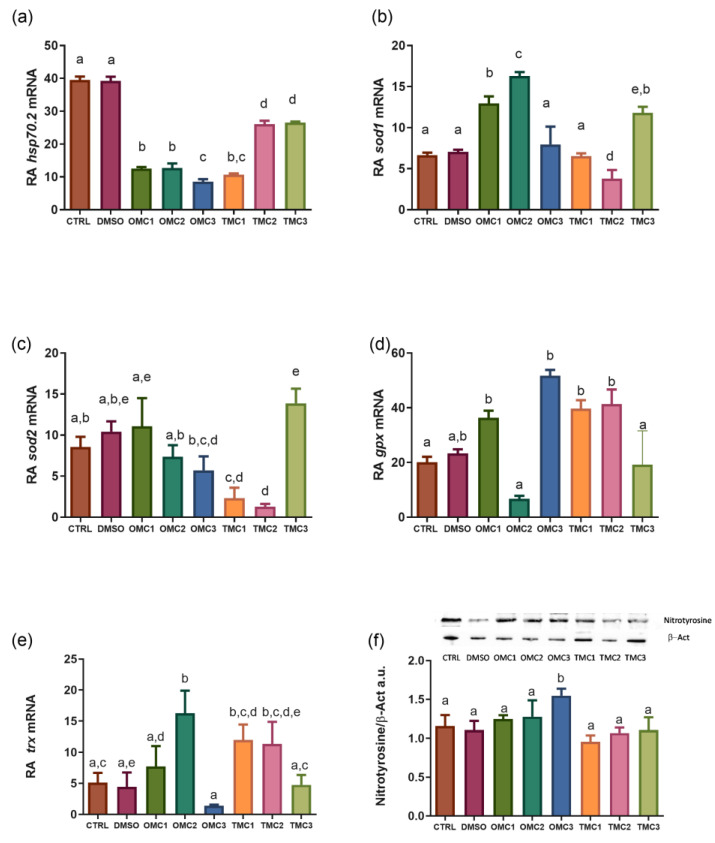
Expression profiles of oxidative stress response biomarkers. (**a**) *hsp70*, (**b**) *sod1*, (**c**) *sod2*, (**d**) *gpx*, and (**e**) *trx* mRNA and (**f**) Nitrotyrosine protein levels in larvae exposed to the different experimental treatments. mRNA levels were normalized against *rplp0* and *rplp13* and used as reference genes. Nitrotyrosine levels were normalized against β-actin. Insert shows representative nitrotyrosine (55 KDa) and b-Act (42 KDa) immunoblots. Data are shown as the mean ± SD. Different letters indicate statistically significant differences among the experimental groups (*p* < 0.05).

By real-time PCR, the expression of Heat shock protein 70 (*hsp70.2*) mRNA, which codifies for a chaperone that helps proteins to maintain their folded structure, was analyzed. The data show a significant decrease in *hsp70.2* mRNA expression in all groups exposed to OMC and TMC compared to the CTRL and DMSO groups. Particularly, the lowest expression of *hsp70.2* was measured in embryos treated with the lowest concentration of OMC (OMC3), and a comparable reduction was observed in OMC1, OMC2, and TMC1-exposed embryos. The medium and lowest TMC concentrations determined a reduction in expression, which was significantly lower with respect to the control groups but higher with respect to that induced in the OMC-treated groups and the TMC1 one (Figure 4a). Regarding the expression of genes codifying for the antioxidant system, superoxide dismutase (*sod*) mRNAs were analyzed. The *Sod1* mRNA level was significantly increased in the OMC1, OMC2, and TMC3 groups compared to the control groups, while a decrease in expression was observed in the TMC2 group (Figure 4b).

Regarding *sod2*, the mRNA transcript increased in TMC3-treated fish. In contrast, a decrease, although not statistically significant with respect to the controls, was observed in OMC2 and OMC3 larvae. Nevertheless, the TMC1 and TMC2 concentrations caused a significant mRNA reduction (Figure 4c). The glutathione peroxidase (*gpx1a*) mRNA levels pinpointed significant differences among the exposed and control groups. Specifically, regarding treatments, in OMC-exposed fish, a U shape response was observed, with an increase induced by the lowest and highest concentrations, while the medium one did not affect the basal levels. Concerning TMC, only TMC1 and TMC2 exposure upregulated mRNA expression, with values similar to those induced by OMC1 and OMC2 concentrations (Figure 4d). Thioredoxin (*Trx*) mRNA expression was significantly upregulated only in OMC2, TMC1, and TMC 2-exposed larvae. Furthermore, in OMC1-treated fish, a slight increase was observed, although not statistically significant (Figure 4e). By a Western blot analysis, the nitrotyrosine (NT) protein levels were assayed. A significant upregulation of the protein levels was observed only in OMC3-treated fish. Noteworthy is the decreasing, although not significant, trend displayed by the TMC treatments (Figure 4f).

### 2.3. Analysis of Molecular Targets Involved in the Endoplasmic Reticulum Stress (ER Stress) Response, Inflammation, and Apoptosis

**Figure 5:**
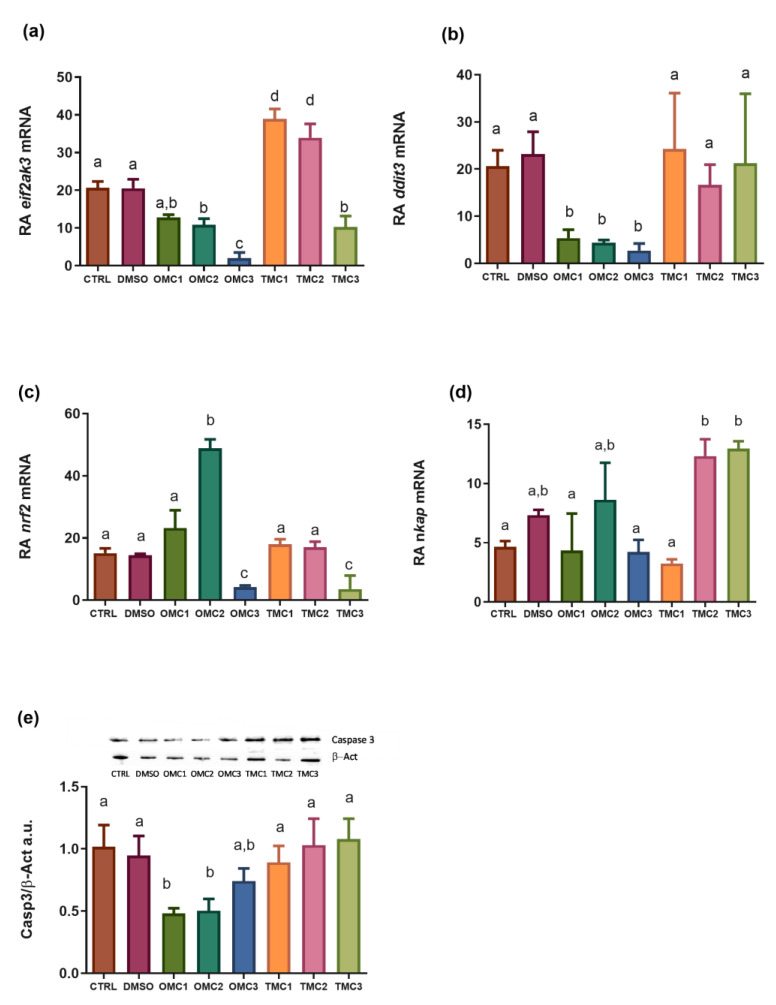
Expression profiles of ER stress biomarkers. (**a**) *eif2ak3*, (**b**) *ddit3*, (**c**) *nrf2*, (**d**) *nkap* mRNA, and (**e**) Casp3 protein levels in larvae exposed to the different experimental treatments. mRNA levels were normalized against *rplp0* and *rplp13* and used as reference genes. Cleaved caspase 3 (Casp3) (17 KDa) levels were normalized against b-actin (b-Act) (42 KDa). Insert (**e**) shows representative Casp3 and β-Act blots. Data are shown as the mean ± SD. Different letters indicate statistically significant differences among the experimental groups (*p* < 0.05).

Among the molecular sensors triggering the onset of ER stress, *eif2ak3* mRNA (codifying for PERK protein) was significantly downregulated in larvae exposed to OMC2, OMC3, and TMC3, while it was upregulated in larvae exposed to TMC1 and TMC2 (Figure 5a). The expression of DNA damage-inducible transcript 3 (*ddit3*), also known as the C/EBP homologous protein (CHOP), significantly decreased in all groups exposed to OMC (Figure 5b). Nuclear receptor factor 2 (*nrf2*) mRNA resulted in significantly upregulated in OMC2 exposed larvae and downregulated OMC3 and TMC3 groups (Figure 5c).

The expression of NF-kB-Activating Protein (*nkap*) mRNA, encoding a protein involved in the activation of the ubiquitous transcription factor NF-kB, was significantly upregulated only in TMC2- and TMC3-exposed embryos (Figure 5d). By a Western blot analysis, the active-cleaved caspase 3 protein levels were assayed and were significantly downregulated only in OMC1 and OMC2-exposed larvae (Figure 5e).

### 2.4. Analysis of Androgen Receptor (ar) Transcript and Estrogen Receptor α (ERα) Protein Levels

**Figure 6:**
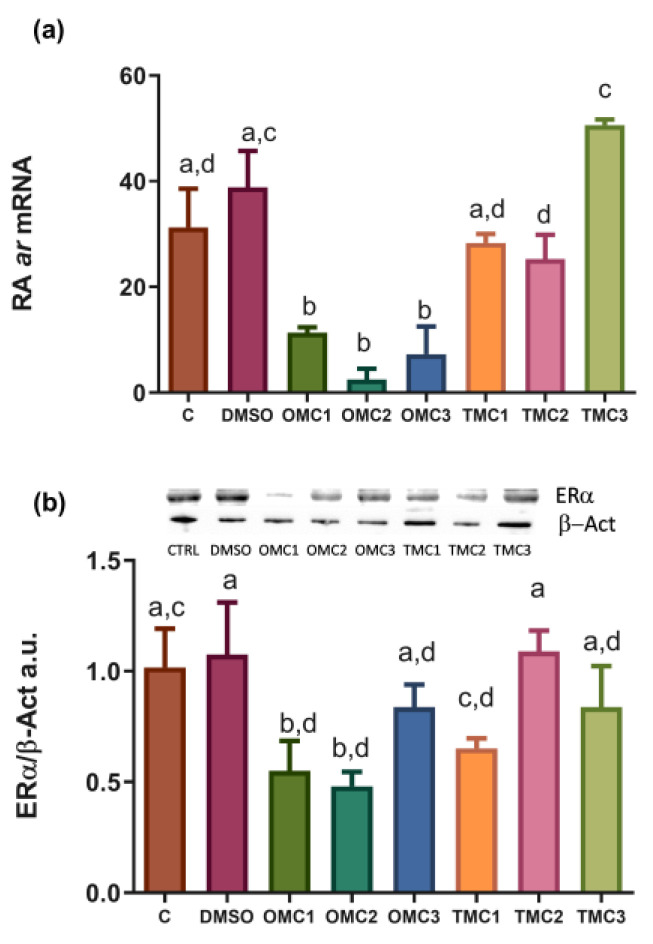
Expression profiles of steroid hormone receptors. (**a**) *ar* mRNA and (**b**) ERα protein levels in larvae exposed to the different experimental treatments. mRNA levels were normalized against rplp0 and rplp13 and used as reference genes. The ERα levels were normalized against β-actin (β-Act). Insert (**b**) shows representative ERα (65 KDa) and β-Act (42 KDa) blots. Data are shown as the mean ± SD. Different letters indicate statistically significant differences among experimental groups (*p* < 0.05).

The *Ar* transcript levels were significantly downregulated by OMC exposure, while the TMC treatment did not induce significant changes either with respect to the DMSO-treated or to CTRL fish (Figure 6a). By a Western blot analysis, the Erα protein levels were analyzed, and a significant reduction was observed in OMC1- and OMC2-treated larvae, while TMC1 exposure caused a significant downregulation only with respect to DMSO-exposed larvae (Figure 6b).

### 2.5. Molecular Docking Results

As reported in the Methodological section, TMC and OMC were docked to estrogen and androgen receptors in order to evaluate their possible interaction and affinity.

**Figure 7:**
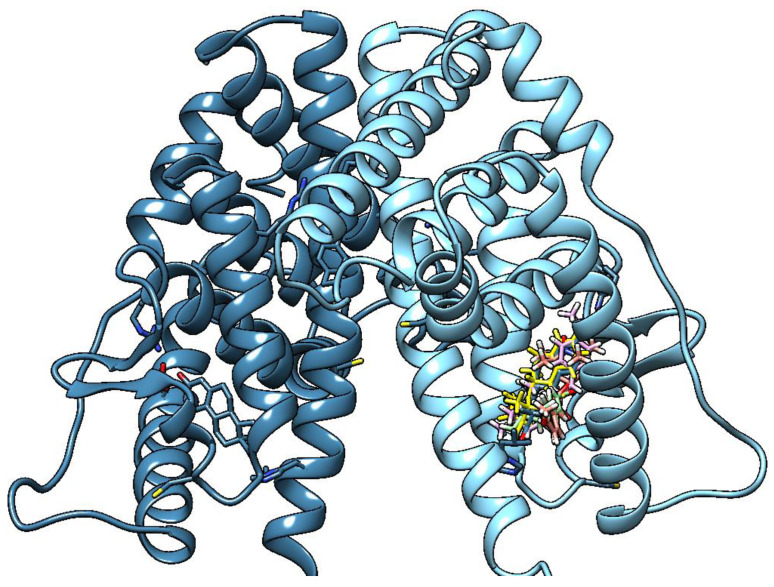
TMC clusters 1–3 (most relevant and significatively populated, tube/sticks model) in the binding cleft of the dimeric estrogen receptor ligand binding domain (LBD) (light blue, chain A/deep blue, chain B) (binding in only one monomer is shown). Estradiol is represented as a yellow tube model.

Estrogenic receptor: for TMC, from the cluster analysis, only one main pose was identified (three clusters; >90% population) located within the estradiol binding cleft (En = −7.74 kcal/mol) (Figure 7). Comparing this positioning with the estradiol and raloxifene ones (derived from crystallographic data 1a52 and 1ere, respectively), we can notice that they are totally superimposable, thus suggesting a competitive binding behavior. However, the estradiol binding energy is much lower (i.e., higher affinity) at −9.68 vs. −7.74 kcal/mol, implying that TMC cannot really compete significatively with the natural ligand (no interference activity).

**Figure 8:**
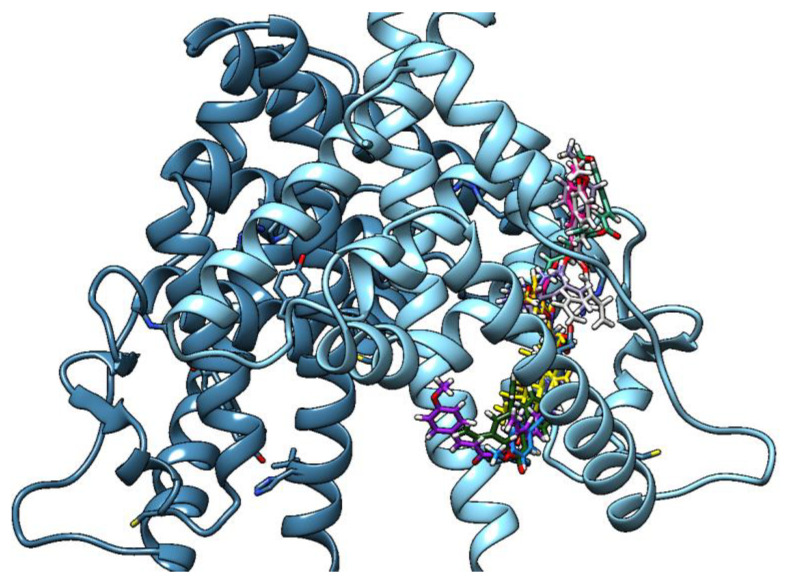
OMC clusters 1–9 (most relevant and significatively populated, tube models) in the binding cleft of the dimeric estrogen receptor LBD (light blue, chain A/deep blue, chain B ribbons) (binding in only one monomer is shown). Estradiol is represented as yellow tubes.

**Figure 9:**
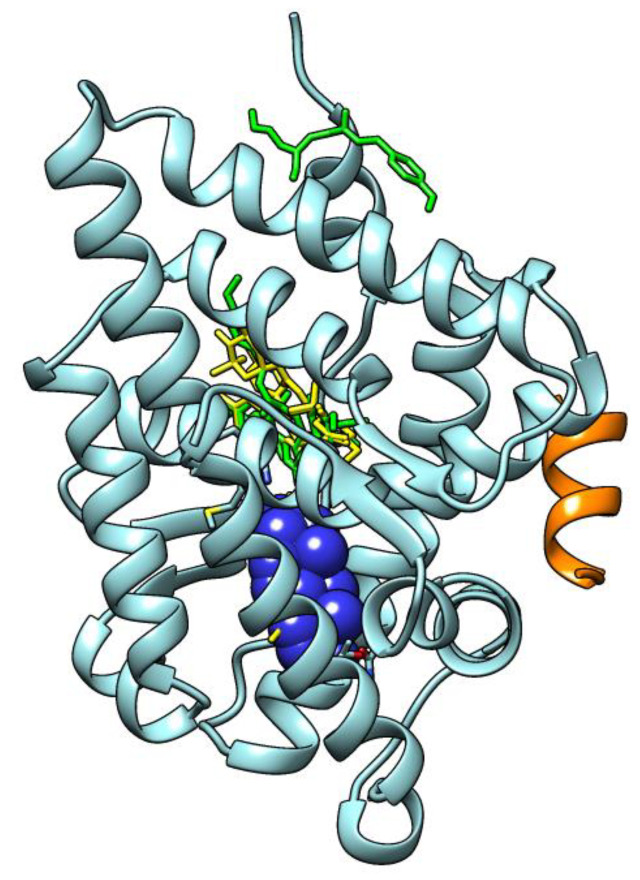
Androgen receptor LBD (cyan, ribbons) in a complex with DHT (dihydrotestosterone, in blue, CPK sphere) and its peptidic coactivator (orange, ribbons); in green tubes, OMC poses; in yellow tubes, TMC ones.

A different result was obtained, however, for OMC: this compound appears to bind more efficiently to the ER, since, from the cluster analysis, 9–10 different orientations were found (all close in energy and significatively populated, range –7.2/−6.80 kcal/mol)) that are spread all along the incoming pathway to the binding cleft (Figure 8). Thus, even if its energy is still higher than that of estradiol within the cleft, the other binding sites, due to their location, will obstruct the natural ligand’s entrance to its cleft. This suggests a possible interference activity. Androgen receptor: For TMC, the cluster analysis after the docking experiments reveals the presence of statistically populated clusters all located at the same site, albeit with different spatial molecular orientations, both far from the natural ligand’s binding cleft and from the coactivator’s (Figure 9). In addition, for OMC (Figure 9), two main poses are identified: one located in the same TMC docking zone, the other located in proximity and bridging two helix regions (aa 843–833 (helix) and 670–677 aa (helix)), which are close to the connection loop of the DNA-binding domain (aa 560–632), not shown in figures. Thus, even if any agonistic activity can be excluded for this compound, the possibility of some allosteric antagonistic activity must still be considered.

## 3. Discussion

In this study, the effects of early exposure to one of the most widely used UV filters, OMC, and one of its derivatives, TMC, were analyzed in zebrafish larvae, focusing on the onset of possible adverse developmental effects. OMC toxicity, in fact, has been largely described in different animal models, and the necessity to replace this molecule has become urgent. TMC, one of its recently synthesized derivatives, could represent a valid candidate molecule, but its effects in vivo have not been investigated so far. For this reason, embryos were exposed to OMC at environmentally relevant concentrations, and the same range was used also for TMC, which, considering its chemical features, could represent a valid, safer alternative UV filter. In this regard, the concentrations of either OMC or TMC used did not induce morphological alterations, resulting in agreement with a previous published paper on OMC [21]. These last authors observed, in fact, that OMC concentrations in the same range as those used in this study, 1 and 10 μg/L, had no effect on the hatching rate, malformation, and survival of zebrafish [21], while 100 μg/L exposure caused developmental toxicity.

Although no significant changes were observed in larval morphometry, evidence was obtained regarding the ability of both compounds to interact with bone formation. In zebrafish, the appearance of the cartilaginous structure can be easily detected 3 or 4 days post-fertilization, and the results obtained herein with the Alcian blue–Alizarin red double staining allowed us to hypothesize that OMC and TMC, similar to other chemical compounds, including metallic elements, pesticides, and drugs [30], interact with bone metabolism and seem to accelerate bone development, suggesting a possible interaction with the endocrine system. Nevertheless, the acceleration in bone formation has been observed not only in the case of xenobiotic exposure but also in the case of probiotic addiction in the rearing medium [31,32,33], suggesting that the presence of the cinnamate moiety in both molecules can boost mineralization.

Most of the studies published to date show that one of the main problems related with exposure to sunlight, as well as to certain UV filters, including OMC, is the increase in oxidative stress [21,34]. The results obtained herein clearly suggest the prooxidant action of OMC, whereas, in the case of TMC, although it still induces the modulation of similar biomarkers to some extent, it is less toxic overall than OMC. Among the analyzed biomarkers, *hsp70* has been largely considered an early warning signal of stress [35,36]. Its primary role, shared with most chaperones, is traditionally linked to protein folding and assembly [37,38] and to the disaggregation of protein aggregates [39]. Later evidence showed that chaperones have a dual function in proteostasis, as they also contribute to key steps in protein degradation [40]. In this light, the downregulation of *hsp70* observed herein suggests an increase in unfolded proteins, which contribute to the unfolded protein response (UPR) onset. Following the translation, secreted and transmembrane proteins enter the lumen of the endoplasmic reticulum (ER), where they are post-translationally modified and properly folded in the presence of specialized chaperone proteins. This process is overwhelmed during ER stress, so, in order to relieve ER stress and restore ER homeostasis, the cell activates UPR, which is regulated by three molecular sensors, one of them being PKR-like ER kinase (PERK). Activated PERK directly acts on eukaryotic translation initiator factor-2 (eIF2a), which reduces the protein overload within the ER of a stressed cell and favors the transcription factor ATF4, which translocates to the nucleus and activates a set of UPR target genes involved in amino acid metabolism, antioxidant responses, autophagy, and apoptosis [41,42]. Among them, CHOP causes a downregulation of Bcl-2 [43], favoring the activation of the intrinsic apoptotic pathway. In this context, in relation to our results, *perk* mRNA, which, in zebrafish, is codified by the *eif2ak3* gene, shows a differential behavior with the two compounds; OMC, in fact, determines its mRNA downregulation, which possibly also causes *ddit3* mRNA, codifying for CHOP, decreased transcription. This signal cascade inhibition can contribute to the reduction in the cleaved, active caspase 3 protein levels and highlights OMC’s ability to interact with larvae physiological processes. It should be considered that, at this stage of development, apoptosis plays a crucial role in correct organ shaping; thus, in the long term, the occurrence of morphological alterations cannot be excluded. On the contrary, *eif2ak3* mRNA is upregulated by the higher TMC concentrations, thus allowing to speculate that, at higher concentrations, the Tempol moiety could exert its beneficial, antioxidant effect and can counteract the toxicity of OMC in these fish. The signaling pathway leading to the activation of apoptosis are, in fact, not induced in TMC-exposed fish, as stated by the lack of changes in the CHOP (*ddit3*) and caspase 3 levels. Nevertheless, PERK (*eif2ak3*) can, in turn, also activate Nrf2, a master regulator of detoxification [44]. Nrf2 is a transcription factor that, once in the nucleus, specifically recognizes and binds to the core sequence of the Antioxidant Responsive genes (AREs). In this context, the increased trend observed in OMC-treated fish, especially at the higher concentrations, for *sod1, txn*, and *gpx* and nitrotyrosine, despite not being always significant, clearly suggest that larvae are undergoing oxidative stress. In addition, since both *sod* mRNA isoforms were not affected by higher TMC concentrations, this further strengthens the hypothesis that TEMPOL probably mitigates OMC toxicity by exerting an antioxidant action. TMC, in fact, only increases the *gpx* and *txn* mRNA levels, and since the GPx/glutathione system is thought to be a major defense in the case of low levels of oxidative stress [45], we can speculate that TMC is less toxic than OMC at this stage of embryogenesis. An increase in *gpx* has also been found in embryos exposed to another UV filter, benzophenone-3, to which chronic exposure, similar to OMC and TMC, did not affect the survival and hatching rates [46,47]. A similar *txn* and *gpx* mRNA trend was observed for both chemicals. In mammalian cell models, it was observed that Trx-1 plays roles in redox regulation, growth promotion, neuroprotection, inflammatory modulation, and the inhibition of apoptosis [48]. In addition, it seems that the glutathione system can serve as a backup system to reduce thioredoxin when the electron transfer pathway from TrxR1 is blocked [49], suggesting a tight crosstalk between the two antioxidant systems. In zebrafish, *txn* knockdown led to hydrocephalus [50] and defective liver development [51], mainly due to increased hepatic cell death.

Regarding *nkap* mRNA, it is known that, aside from being directly involved in NF-kB activation, a master gene in inflammatory processes [52], it has a key role also in transcriptional repression, immune cell development, maturation, T-cell acquisition of functional competency, and the maintenance of hematopoiesis [53]. Nevertheless, a role of NF-kB in immune system development has also been described, resulting in a major transcription factor that regulates the genes responsible for both the innate and adaptative immune response. Thus, the increase of this transcript in TMC-exposed larvae could be directly associated with a more enhanced immune response, which correlates well with the more developed skeletal structure, as pointed by morphological observations.

**Figure 10:**
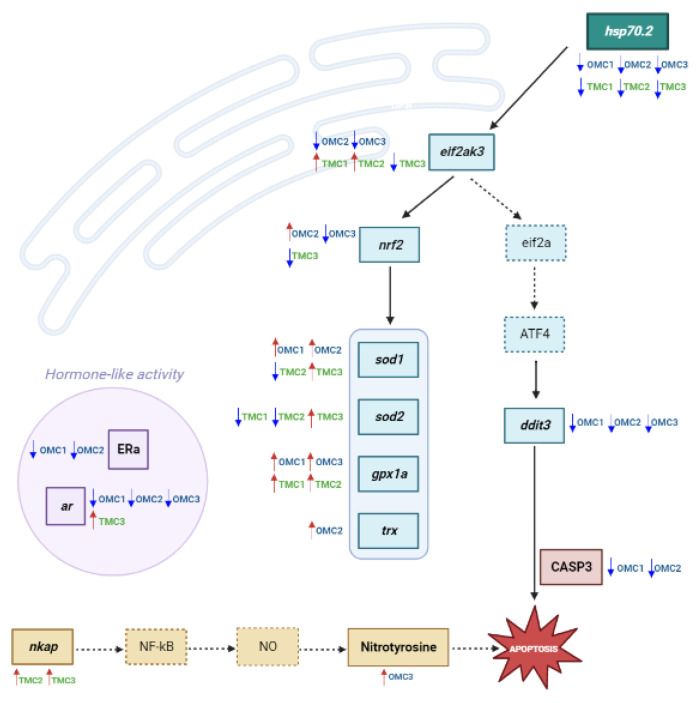
Graph chart showing the analyzed biomarkers’ network. Analyzed targets are marked in bold within boxes, while those in dotted boxes were not analyzed. Arrows indicate significantly increased (⬆) or decreased (⬇) levels/expression with respect to the control ones. eif2a, Eukaryotic translation initiation factor 2A; ATF4, Activating Transcription Factor 4; NO, nitric oxide.

Finally, the first attempt to investigate the possible hormone-like activity of TMC was carried out. Our results suggest that TMC does not affect the *ar* transcription nor ERα protein levels, ruling out a possible hormone-like activity. These findings are supported from the in silico docking studies carried out on both the ER and AR receptors; in fact, in relation to ER activity, TMC cannot compete with estradiol due to its lower affinity and the absence of allosteric sites of interactions. Regarding OMC, different studies so far have demonstrated its ability to interact with steroid hormone receptors; in a yeast assay [54] and in vitro using MCF-7 cells (E SCREEN), OMC behaved as an estrogen-like compound upregulating the ER levels. Our in silico studies showed, for OMC, the presence of allosteric sites of interactions that prevent entrance of the natural ligand in its binding cleft. In addition, considering OMC with AR, the presence of a possible allosteric site at the beginning of the connection loop with the DNA-binding site could suggest the possible inhibition of AR activity. This site is instead absent for TMC. Further evidence with hormone receptors comes from studies in vivo in rats, where OMC increased the uterine weight [55]. A different scenario was obtained in a study using zebrafish reporting the ability of OMC to inhibit *er* isoform transcription [21], thus resulting in agreement with the results herein obtained. This variety in the response should be considered when working with in vivo models that could exhibit a different tolerance to xenobiotics; thus, the same molecule, the specific concentration used, and the length of exposure could act on a species-specific basis. The results obtained in this study are summarized in Figure 10. This schematic figure shows that TMC, differently from OMC, which significantly affects both the *ar* and ERα levels, has scarce hormone-like activity. Only OMC affects apoptosis, which has a key role in embryo shaping at this stage of early development, and finally, TMC potentiates the larvae oxidative stress response, suggesting that organisms could be more prone to contrasting ROS production caused by oxidative stimuli, such as UV light exposure.

## 4. Materials and Methods

### 4.1. Chemicals

OMC (98%), TEMPOL (97%), *p*-methoxy cinnamic acid (99%), *N*,*N*-dicyclohexylcarbodiimide (DCC) (99%), and 4-dimethylaminopyridine (DMAP) (99%), as well as all the other reagents and solvents, were purchased from Sigma-Aldrich and were used without further purification. TMC was synthesized starting from *p*-methoxy cinnamic acid and TEMPOL by DCC/DMAP-mediated esterification: *p*-methoxy cinnamic acid (1 mmol), DMAP (0.05 mmol), and TEMPOL (1 mmol) were dissolved in 2 mL of anhydrous dichloromethane (CH_2_Cl_2_) and cooled to 0 °C; 1.2 mmol of DCC were then added and magnetically stirred for 5 min at 0 °C. The reaction mixture was left for 3 h at room temperature under stirring. The reaction course was monitored by thin-layer chromatography by using petroleum ether/diethyl ether (8:2) as the eluant. At the end of the reaction, the insoluble *N,N*-dicyclohexylurea was filtered off, and the filtrate was evaporated. The residue was dissolved in CH_2_Cl_2_ and washed with saturated NaHCO_3_ (3 × 10 mL). The organic layer was dried over Na_2_SO_4_ anhydrous, and the solvent was removed under reduced pressure, affording a 70% yield of TMC. The purity of the compound was assessed by comparison with an authentic sample, as described in [24].

### 4.2. Exposure

Embryos were obtained by natural spawning by crossing adult zebrafish (*D. rerio*, AB wild-type strain) maintained at the DiSVA fish facility under controlled conditions (28.0 ± 0.5 °C) and with a 14/10 h light/dark cycle in oxygenated water. Embryos were obtained by natural spawning crossing 10 couples. Spawned embryos were reared in E3 medium (5 mM NaCl, 0.17 mM KCl, 0.33 mM CaCl_2_, 0.33 mM MgSO_4_, and 10–5% Methylene Blue). All spawned eggs were checked for fertilization and quality and then were divided among experimental groups in 250 mL glass beakers and were maintained at 1 embryo/mL stocking density (150 embryos/beaker). Each experimental group was set up in triplicate. The experimental groups were:

- Control;
- DMSO: exposed to 0.01% DMSO (vehicle);
- OMC1: exposed to 0.05 μM OMC;
- OMC2: exposed to 0.01 μM OMC;
- OMC3: exposed to 0.005 μM OMC;
- TMC1: exposed to 0.05 μM TMC;
- TMC2: exposed to 0.01 μM TMC;
- TMC3: exposed to 0.005 μM TMC.

OMC and TMC were dissolved in DMSO. The exposure started immediately after the embryo viability was checked and lasted until 96 h post-fertilization (hpf). The fish rearing medium was changed daily, and DMSO, OMC, and TMC were renewed. After 96 h, at least 10 individuals randomly taken from each group were sacrificed, fixed in 4% PFA, and stored in 70% ethanol. The other larvae were collected and stored at −80 °C for molecular analyses.

### 4.3. Morphology

All embryos stored in ethanol 70% were observed and photographed with a stereomicroscope (Leica microsystems, Wetzlar, Germany). The following parameters were recorded: total length, eye length, yolk area, and yolk circumference.

### 4.4. Alcian Blue–Alizarin Red Staining

Five larvae per experimental group were double-stained according to Maradonna et al. 2013 [31]. Larvae were then observed and photographed with a Zeiss Axio Imager.A2 combined with a color digital camera Axiocam 105 (both from Zeiss, Oberkochen, Germany) optical microscope. Images were analyzed using ZEN 2.3 (Carl Zeiss Microscopy GmbH, Oberkochen, Germany).

### 4.5. RNA Extraction and cDNA Synthesis

Total RNA was extracted, and cDNA synthesis was performed as previously described in [56], starting from 5 pools of ±20 larvae for each experimental group. Further details are reported in the Supplementary Materials.

### 4.6. Real-Time PCR

The qRT-PCRs were performed using the dye-based SYBR green assay in a CFX thermal cycler (Bio-Rad, Milan, Italy), as previously described in [56]. For each experimental group, replicates (*n* = 5) were run in duplicate. Primer list is reported in Supplementary Table S1 and further details are reported in the Supplementary Materials.

### 4.7. Western Blot Analysis

Whole embryo homogenates were extracted from at least 4 pools of ±40 larvae. Protein extraction, SDS page electrophoresis, and Western blot procedures are detailed in the Supplementary Materials.

### 4.8. Computational Analysis

Estrogen and androgen receptor LBD (ligand-binding domain) structures were retrieved from the Brookhaven Protein Data Bank (PDB codes 1ere/1a52 and 1e3g/1t5z, respectively) “http://www. wwpdb.org (accessed on 24/02/2022)” and used in the molecular docking calculations. TMC and OMC structures were built in and optimized at the DFT level using the B3LYP/6–311G ** basis set. Mulliken charges were calculated, then kept for subsequent docking simulations with AutoDock 4.2/MGLTools 1.5.7 [57]. For both compounds, a blind docking approach was used at first in order to identify after the cluster analysis every putative site on the receptor’s surface. Subsequently, on the lowest energy and most populated poses, a focused docking protocol was applied to better refine both pose and its energy. For the blind docking, the grid map, centered in the center of mass of the enzyme (120 × 120 × 120 Å^3^), included the whole protein surface; in the focused docking protocol, the grid map was centered on the ligand and extended around the cleft (40 × 40 × 40 Å^3^) with points spaced equally at 0.375 Å. The number of GA (genetic algorithm) runs was set to 100, the energy evaluations 25,000,000, the maximum number of top individuals that automatically survive 0.1, and the step size for translation 0.2 Å. All the docking calculations were carried out in triplicate using three different CPU random seeds. The final docked ligand–receptor complexes were ranked according to the predicted binding energy, and all the conformations were processed using the built-in clustering analysis with a 2.0 Å cut-off. Additionally, for purposes of comparison, estradiol was docked to the estrogen receptor LBD in order to compare the TMC/OMC-binding energies. For AR, the DHT (dihydro testosterone) binding site was taken into account in order to locate the TMC/OMC binding zones (1t5z PDB code). Molecular graphics images were produced using the UCSF Chimera 1.16 package (Resource for Biocomputing, Visualization, and Informatics at the University of California, San Francisco, CA, USA).

### 4.9. Statistical Analysis

QPCR, Western blot, and the imaging statistical analysis were performed with Graph Pad Prism V9.0.1. (GraphPad Software, Inc., San Diego, CA, USA). Data were presented as the means ± SD and were analyzed using one-way ANOVA, followed by Dunnett’s multiple comparison test. Different letters on the histogram bars indicate statistically significant changes among the groups. The *p*-value was set as *p* < 0.05.

## 5. Conclusions

In conclusion, the results obtained during this in vivo study of TMC, a derivative of the most popular UVB filter, OMC, are promising, as it appears to be less toxic than its parent derivative. The integration of the results suggests that TMC could replace OMC in the future as an equally effective photoprotectant but endowed with less toxic effects. However, additional in vitro studies or in vivo ones using other marine species would help in supporting the preliminary data obtained here and would aid in better understanding the behavior of this promising compound.

## Figures and Tables

**Table 1:** The table shows representative images of 48 hpf embryos exposed to DMSO (vehicle control) and to the 3 different concentrations of OMC and TMC (1 = 0.05 mM, 2 = 0.01 mM, and 3 = 0.005 mM). The morphological parameters, as shown in Figure 1, are reported. D1—Eye Diameter 1 (µm); D2—Eye Diameter 2 (µm); D.B.E.—Distance between eyes (µm); E.D.—Eye diameter (µm).

48 hpf	Rappresentative Image	D1	D2	E.D.	D.B.E.
CTRL	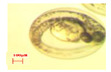	1198.36 ± 84.77	1183.15 ± 91.31	412.09 ± 58.31	85.33 ± 23.15
DMSO	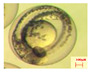	1168.3 ± 40.30	1141.65 ± 42.22	324 ± 30.48	60 ± 28.28
OMC1	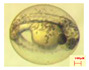	1195.83 ± 44.59	1167.75 ± 49.60	368.33 ± 73.54	98 ± 47.73
OMC2	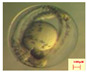	1196.08 ± 56.72	1151 ± 56.67	318 ± 65.49	72.23 ± 18.32
OMC3	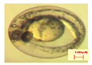	1185.53 ± 46.44	1164.8 6± 48.39	342.5 ± 29.89	48.33 ± 18.58
TMC1	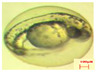	1199.91 ± 85.44	1167.58 ± 74.16	299.14 ± 51.19	47.66 ± 7.57
TMC2	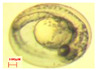	1167 ± 40.12	1130.23 ± 60.33	305.22 ± 36.71	65.66 ± 27.06
TMC3	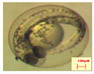	1196.07 ± 58.46	1214.21 ± 223.72	345.07 ± 37.13	49 ± 11.04

**Table 2:** The table shows representative images of 72 hpf larvae exposed to DMSO (vehicle control) and to the 3 different concentrations of OMC and TMC (1 = 0.05 mM, 2 = 0.01 mM, and 3 = 0.005 mM). The morphological parameters, as shown in Figure 1, are reported. L—larval length (µm), E.L.—eye length (µm), Y.A.—yolk area (µm^2^), and Y.P.—yolk perimeter (µm).

72 hpf	Rappresentative Image	L	E.L.	Y.A.	Y.P.
CTRL	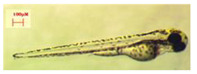	3611.66 ± 260.67	271 ± 32.03	174,437 ± 28,116.37	1774.88 ± 140.17
DMSO	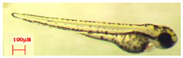	3490.11 ± 298.73	249.11 ± 40.58	193,376.44 ± 29,491.53	1859 ± 127.52
OMC1	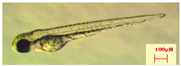	3571.55 ± 146.17	261 ± 19.41	210,217.66 ± 31,725.03	1763.77 ± 586.06
OMC2	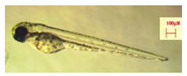	3576.33 ± 121.05	254.22 ± 20.85	172,466.88 ± 26,570.47	1757.66 ± 128.10
OMC3	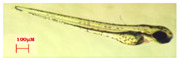	3422.88 ± 253.89	248.66 ± 30.62	199,967.77 ± 34,357.39	1876.44 ± 139.70
TMC1	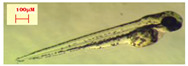	3385.88 ± 339.38	258.33 ± 36.77	207,813.22 ± 38,902.59	1928.55 ± 184.00
TMC2	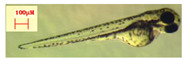	3442.33 ± 244.32	259.33 ± 23.61	212,495.33 ± 41,906.682	1947 ± 192.24
TMC3	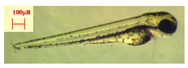	3549.22 ± 244.46	251.33 ± 43.82	185,205.77 ± 28,941.01	1882.55 ±178.39

**Table 3:** The table shows representative images of 96 hpf larvae exposed to DMSO (vehicle control) and to the 3 different concentrations of OMC and TMC (1 = 0.05 mM, 2 = 0.01 mM, and 3 = 0.005 mM). The morphological parameters, as shown in Figure 1, are reported. L—larval length (µm), E.L.—eye length (µm), Y.A.—yolk area (µm^2^), and Y.P.—yolk perimeter (µm).

96 h	Rappresentative Image	L.	E.L	Y.A.	Y.P.
CTRL	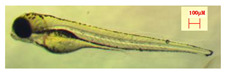	3735.66 ± 473.19	283.4 ± 41.01	189,534.14 ± 3420.9	1861.14 ± 141.42
DMSO	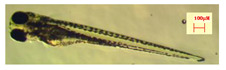	3751.07 ± 218.15	295 ± 20.91	183,176.21 ± 34,651.49	1865.57 ± 180.09
OMC1	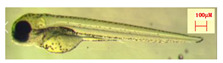	3713.06 ± 324.75	282.4 ± 23.46	189,039.33 ± 29,766.13	1916.53 ± 98.61
OMC2	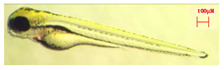	3712.73 ± 287.94	291.93 ± 37.82	204,543.13 ± 34,860.92	1965.6 ± 136.70
OMC3	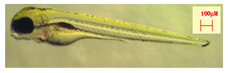	3733.6 ± 263.70	287.53 ± 32.42	196,972.66 ± 26,756.82	1932.86 ± 120.59
TMC1	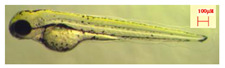	3714.2 ± 359.77	279.66 ± 41.19	216,675.4 ± 42,217.47	2019.86 ± 186.53
TMC2	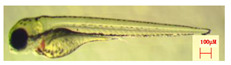	3767.4 ± 271.57	280.46 ± 18.65	187,298.93 ± 29,537.75	1892.8 ± 125.72
TMC3	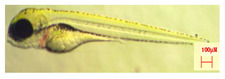	3771.4 ± 291.18	285.73 ± 23.15	206,535.73 ± 24,249.74	1995.86 ± 112.04

## Data Availability

The data presented in this study are available on request from the corresponding author.

## Supplementary Materials

The supporting information can be downloaded at: https://www.mdpi.com/article/10.3390/ijms24076767/s1.
