# Supplementary material for: First In Vivo Insights on the Effects of Tempol-Methoxycinnamate, a New UV Filter, as Alternative to Octyl Methoxycinnamate, on Zebrafish Early Development
Source: Int J Mol Sci. 2023 Apr 5;24(7):6767. doi: 10.3390/ijms24076767 (PMC10094805; doi:10.3390/ijms24076767)
Supplement: Supplementary file 1 [file ijms-24-06767-s001.zip › ijms-2277177-supplementary.pdf]

Supplementary Material to Damiani et al.,

RNA extraction and cDNA synthesis

RNA extraction was performed using RNAzol RT (Merck KGaA, Darmstad, Germany, # R4533). Genomic DNA was removed by DNase I digestion (Merck KGaA, Darmstad, Germany, # AMPD1). RNA concentrations were determined by nanophotometer P330 (Implen, München, Germany) and integrity was assayed by 1% gel electrophoresis. A total amount of 1 µg RNA was used for cDNA synthesis with iScript cDNA Synthesis Kit (Bio-Rad, Milano, Italy).

Real-time PCR

Final primer concentration was 10 pmol/µL. Ribosomal protein 13 (*rpl13*) and ribosomal protein 0 (*rpl0*) mRNAs were used to normalize target gene expression levels analysed by CFX Manager Software version 3.1 (Bio-Rad), including GeneEx Macro Conversion and GenEx Macro files and results are represented by bar-plots along with the standard deviation. Specific primer pairs for target genes were designed with Primer-Blast.

Supplementary Table S1. Primer list.

Gene Acronym	NCBI gene accession no.	Forward	Reverse
rpl13a	NM_212784.1	TCTGGAGGACTGTAAGAGGTATGC	AGACGCACAATCTTGAGAGCAG
rplp0	NM_131580.2	CTGAACATCTCGCCCTTCTC	TAGCCGATCTGCAGACACAC
hsp70.2	NM_001362360.1	AAAGCACTGAGGGACGCTAA	TGTTCAGTTCTCTGCCGTTG
sod1	NM_131294.1	GTCGTCTGGCTTGTGGAGTG	TGTCAGCGGGCTAGTGCTT
sod2	NM_199976.1	CCGGACTATGTTAAGGCCATCT	ACACTCGGTTGCTCTCTTTTCTCT
gpx1a	NM_001007281.2	GAA GGT GGATGT GAATGG AAA(A,G)GATG	CTG ACG GGA CTC CAA ATG ATG (G,T)
trx	NM_001002461.1	AGACCATCGGGCCGTACTTT	GGCCACATCCTGTGCATCAT
eif2ak3	XM_021480766.1	TGGGCTCTGAAGAGTTCGAT	TGTGAGCCTTCTCCGTCTTT
ddit3	NM_001082825.1 XM_005166171.4	AAGGAAAGTGCAGGAGCTGA	TCACGCTCTCCACAAGAAGA
nrf2a	NM_212855.2	TTTGGTTCCCGATGAAGACG	ACCCAATAGATCTACAGAGC
nkap	NM_001003414.1	AGAGAGCGCTTGCGTCCTT	TTGCCTTTGGTTTTTCGGTAA
ar	NM_001083123.1	ACTGGGACCGAATAAAGCCC	ATGTAATCGCAGCCGGAGAC

Western blot analysis

Whole embryo homogenates were extracted by homogenizing in Lysis buffer (0.125 M Tris,4% (p/v) SDS, 20% (v/v) glycerol, 10% (v/v) β-mercapto-ethanol) and by adding anti-proteolytic (Sigmafast, Merck KGaA, Darmstadt, Germania). Proteins extracts were quantified by absorbance spectroscopy at 595 nm using the Pierce™ Coomassie Plus (Bradford) Assay Kit (Invitrogen). SDS-page was performed on a 4% stacking gel and 12% running gel at 30 mA for 30 min and then at 60 mA until desired migration of samples. The transfer was made on a nitrocellulose membrane with the Bio-Rad mini trans-blot, following customized protocols. Blots were blocked for 1 h at room temperature with a blocking solution containing TBS, BSA 5% (p/v) and 0.1% Tween-20. The incubation with the primary antibody, Nitrotyrosine (GeneTex, Irvine, CA, USA, clone 39B6, #GTX41979), Caspase 3 (Cell Signaling, Beverly, MA, USA, # BK9661S), β-ACTIN (Cell Signaling, Beverly, MA, USA, #4967), estrogen receptor (Sigma-Aldrich, St- Louis, Missouri, USA, #07662) was made overnight at 4 °C followed by incubation with the secondary antibody for 1 h at room temperature. After incubation, blots were

washed with a solution containing TBS and 0.2% (v/v) Tween-20, three times for 10 min. The reaction was visualized with ECL-PLUS (GE Healthcare, Milano, Italy) chemiluminescent reagent for Western blotting. Densitometric analysis was performed using Fiji software for Windows.
